# Physiologic and molecular responses of *indica–japonica* subspecies tetraploid rice seed germination to ion beams

**DOI:** 10.1038/s41598-022-22887-6

**Published:** 2022-10-25

**Authors:** Yaqin Huang, Jinzhe Li, Qunce Huang

**Affiliations:** 1 Xinyang Agriculture and Forestry University, No. 1 Beihuan Road, Pingqiao District, Xinyang City, 464000 Henan Province China; 2 Henan Key Laboratory of Ion-Beam Bioengineering, School of Physics (School of Microelectronics), Zhengzhou University, Zhengzhou, 450052 China

**Keywords:** Biochemistry, Biological techniques, Biophysics, Molecular biology

## Abstract

Ionizing radiation can not only reduce the yield of rice but also cause rice toxicity, and consumption of this kind of rice threatens human health. Moreover, the production and application of freon has further caused a hole in the earth’s ozone layer, increasing the amount of ionizing radiation from the sun affecting rice. To select and breed new radiation-resistant rice varieties, dry seeds of the *indica*–*japonica* subspecies of tetraploid rice subjected to different doses of ionizing radiation were investigated for their responses during germination. The results showed that the relative water absorption, seed vigour and GA_3_ content sharply decreased in response to three different doses of ionizing radiation, and the regulation of the expression of genes related to α-amylase synthesis and gibberellin metabolism was disrupted. Moreover, the degree of inhibition increased with increasing dose. Notably, under 3.0 × 10^17^ ions/cm^2^ radiation, an upregulation of *OsGA3ox2* expression resulted in a sharp increase in GA_3_ content in the *indica–japonica* tetraploid rice, and upregulated expression of *OsAmy3A* and *OsAmy3D* resulted in sharp increase in α-amylase activity, water absorption, and sucrose and fructose contents, which resulted in the seed vigour being greater than that of its parents. The results indicate that additional research on the physiological and molecular features of *indica–japonica* tetraploid rice seed germination in response to ionizing radiation is needed.

## Introduction

Mutation breeding is an effective method for developing new biological resources for plants and microorganisms. More than 3000 mutant varieties have been officially registered with the FAO/IAEA. Most of these mutants were treated with ionizing radiation^1^. Ion beams are essential sources of ionizing radiation. Ion beams are characterized by inducing mutations at a high frequency and inducing a broad spectrum of mutations. The mutation induction frequency from ions is 20 times higher than that of electrons^2^. When rice was irradiated with ions, various chlorophyll mutations (Albina, Xantha, and Viridis) were observed in the M_2_ population^3^. Single-nucleotide variations and insertion/deletions and structural variations can be generated in rice owing to the high linear energy transfer (LET) characteristics of ionizing radiation^4^. LET-responsive regulatory genes were also found, and their expression levels increased with increasing LET values. Among the LET-responsive upregulated genes, *OsPARP3* and *OsPCNA*, which are involved in the DNA repair pathway, have been reported^5^. Genes involved in cell regulation and signal transduction, information storage and processing, and metabolic regulation have also been shown to be induced in response to ionizing radiation^6^. Ion beams can induce high expression of genes related to abscisic acid (ABA) biosynthesis in rice, directly leading to an increase in endogenous ABA contents^7^ The free radicals produced by ion bombardment promote antioxidant enzyme activity and increase lipid peroxidation levels^8–10^. Dose-dependent damage effects have been observed in the M_1_ generation developed from *indica* and *japonica* rice lines^11^.

Polyploidization plays a vital role in plant evolution. Polyploidization is an effective way through which the genetic diversity of plants and heterosis can increase more so than that of diploids. Moreover, autotetraploid varieties have more significant heterosis and genetic diversity than do diploid varieties^12–15^. *Indica* and *japonica* autotetraploid hybrid plants produced by colchicine-induced doubling are large, which provides support for increased yields^16^. Compared with diploid rice, Aautotetraploid rice has a higher photosynthetic capacity and light energy use efficiency and more effectively converts photosynthetic products into starch^17^. Chromosome doubling of rice can increase tolerance to drought stress^18^, and chromosome polyploidization induces the expression of genes related to pollen ontogeny, cell metabolism, cell physiology and so forth^19^. Many essential genes, including meiosis-related, sugar metabolism-related and starch synthase-related genes, have revealed heterosis-specific expression modes in polyploid rice^20^.

N^+^ beam irradiation can alter many characteristics of rice, but there are apparent differences in various ploidy lines and various individual plants of the same line^21^. An appropriate dose of ionizing radiation can induce changes in the activity of antioxidant enzymes and esterase, reduce the accumulation of malondialdehyde (MDA) and help maintain typical membrane structure and metabolism^22^. *Indica* and *japonica* are two major cultivated subspecies of rice^23^. In general, the heterosis of progeny resulting from intersubspecific crosses is more visible than that of progeny resulting from intrasubspecific crosses^24^. Compared with *indica–japonica* diploid rice hybrids, *indica–japonica* tetraploid rice hybrids subjected to ionizing radiation present more advantages than their individual parents and more competitive heterotic effects^25^.

Most physiological and biochemical indexes of rice are correlated with seed germination rate and vigour index, especially soluble protein and catalase (CAT) contents^26^. As rice seeds develop, the contents of soluble sugars and total starch increase markedly^27^. Gibberellic acid 3 (GA_3_) promotes the germination of upland rice seeds under drought stress due to an increase in the expression of α-amylase and expansin genes^28^. Gibberellic acid-responsive genes, ABA-responsive genes, α-amylase and β-amylase are markedly upregulated in the early phase of seed germination^29^.

This study was carried out to investigate the physiological and molecular theories of seed germination of *indica–japonica* tetraploid rice subjected to ionizing radiation. Dry rice seeds were irradiated with various doses of a beam of N^+^ ions. Taking the *japonica* female parent and *indica* male parent as control materials, we analysed the seed water absorption, seed vigour, seedling growth index, α-amylase activity, soluble protein content and carbohydrate content, as well as the expression of pivotal genes involved in α-amylase and gibberellin metabolism.

## Results

### Effects of ionizing radiation on relative water absorption

**Figure 1:**
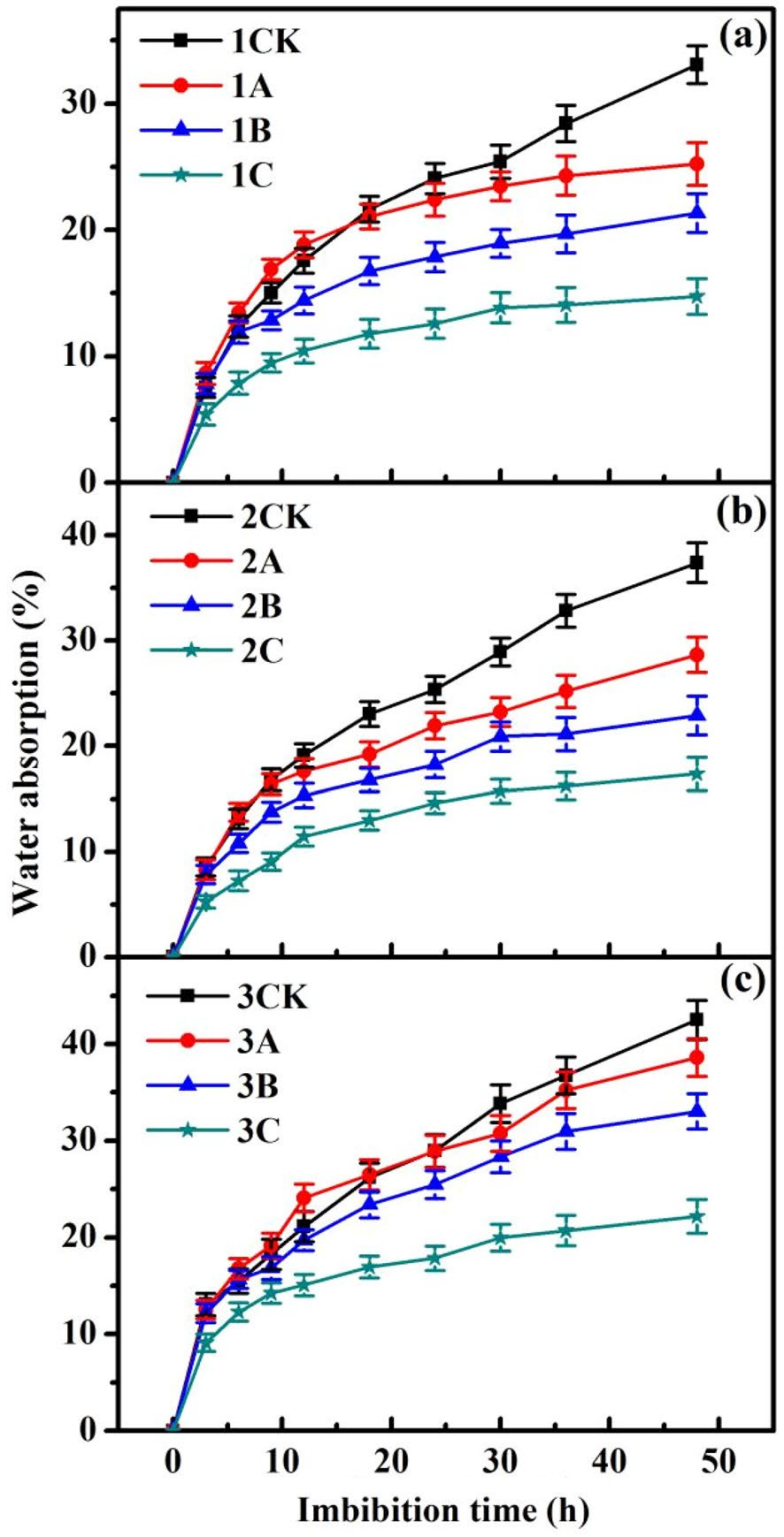
Relative water absorption of rice seeds as a function of time in (**a**) XD10-01 (♀), (**b**) BX10 (♂), and (**c**) XJ (04)-08 subjected to different doses of N ion beam irradiation. XD10-01 (♀), BX10 (♂), and XJ(04)-08 are numbered 1, 2, and 3, respectively. The four ionizing radiation doses, namely, 0 × 10^17^, 3.0 × 10^17^, 5.0 × 10^17^, and 7.0 × 10^17^ ions/cm^2^, of each experimental material are labelled CK, A, B, and C, respectively. The means (± SEs) were calculated from three replicates per treatment. The same scheme applies to the figures below.

Water is critical during the process of seed germination. Nutrients stored in cotyledons or endosperm are translocated as water is absorbed by embryos. As shown in Fig. 1, the water absorption of rice seeds was affected by ionizing radiation. Moreover, the results show that for the diploid parents and the tetraploid rice, the larger ionizing radiation dose was, the greater the inhibition of water absorption, which is a typical ionizing radiation dose effect.

In addition, the characteristics of the seed water absorption curves of the different rice materials subjected to different doses of ionizing radiation varied. Figure 1 shows that after low-dose irradiation, the seed water absorption rate of the tetraploid rice showed no significant difference compared with that of the control rice within 0–36 h and decreased by 9.1% compared with that of the control rice after 48 h (3A). Moreover, the seed water absorption rate of the female parent was not markedly different from that of the control rice within 0–18 h, decreased compared with that of the control rice within 18–48 h, and decreased by 23.7% compared with that of the control rice after 48 h (1A). Furthermore, the seed water absorption rate of the male parent was not markedly different from the control rice within 0–12 h, decreased compared with that of the control rice within 12–48 h, and decreased by 23.4% compared with the control rice after 48 h (2A).

### Effects of ionizing radiation on seed vigour and seedling growth indexes

The germination vigour of rice seeds and the growth of seedlings after ionizing radiation were markedly inhibited (Table 1). Table 1 shows that the seed vigour and seedling growth indexes of the tetraploid rice and its parents tended to gradually decrease with increasing radiation dose, and the six indexes of the high-dose irradiation groups (1C, 2C, 3C) all decreased markedly (*P* < 0.01). Notably, there were also differences in six indexes in response to the ionizing radiation, among which the germination rate, germination index, vigour index and seedling height showed a significant response to low-dose irradiation, while the root number and root length did not change significantly.

**Table 1:** Seed vigour and seedling growth indexes of rice subjected to different doses of N ion beam irradiation.

Treatment group	Germination rate (%)	Germination index	Vigour index	Seedling height (cm)	Root number	Root length (cm)
1CK	94.83 ± 0.58	42.97 ± 0.47	11.88 ± 0.52	3.97 ± 0.34	3.24 ± 0.39	3.06 ± 0.54
1A	89.84 ± 0.55*	35.71 ± 0.53**	9.76 ± 0.44**	3.71 ± 0.21*	3.09 ± 0.49	2.96 ± 0.41
1B	66.19 ± 0.42**	29.48 ± 0.66**	6.13 ± 0.39**	3.4 ± 0.38**	2.97 ± 0.28*	2.78 ± 0.32*
1C	27.31 ± 0.76**	21.82 ± 0.49**	4.87 ± 0.48**	3.06 ± 0.23**	2.74 ± 0.33**	2.34 ± 0.39**
2CK	99.52 ± 0.93	38.55 ± 0.87	13.75 ± 0.98	4.22 ± 0.17	3.45 ± 0.5	3.32 ± 0.44
2A	91.43 ± 1.08*	33.16 ± 0.78**	10.07 ± 0.81**	3.95 ± 0.14*	3.28 ± 0.61	3.18 ± 0.5
2B	55.73 ± 0.34**	24.87 ± 0.73**	8.73 ± 0.64**	3.61 ± 0.22**	3.11 ± 0.57*	3.11 ± 0.26*
2C	36.27 ± 0.48**	19.36 ± 0.52**	5.99 ± 0.42**	3.26 ± 0.15**	2.86 ± 0.63**	2.87 ± 0.62**
3CK	93.94 ± 0.61	39.88 ± 0.69	22.63 ± 0.86	4.53 ± 0.24	3.87 ± 0.64	3.36 ± 0.47
3A	90.26 ± 0.46	36.45 ± 0.59*	19.54 ± 0.76**	4.39 ± 0.18	3.76 ± 0.79	3.22 ± 0.33
3B	82.17 ± 0.39**	35.98 ± 0.87*	16.41 ± 0.62**	3.93 ± 0.15**	3.69 ± 0.74	3.09 ± 0.28
3C	32.39 ± 0.35**	22.93 ± 0.57**	8.56 ± 0.57**	3.55 ± 0.11**	3.38 ± 0.66**	3.01 ± 0.36**

The values are presented as the mean ± SE (n = 3). * and ** denote significance at the *P* < 0.05 and *P* < 0.01 levels, respectively, compared with the control group.

Otherwise, the response of the tetraploid rice to ionizing radiation was markedly lower than that of its parents. Compared with those of the control rice (3CK), the germination rate, seedling height, root number and root length of the tetraploid rice decreased statistically insignificantly (*P* > 0.05), and the vigour index decreased by 13.7% (*P* < 0.01) (3A). However, the germination rate, germination index, vigour index and seedling height of the parents decreased markedly, among which the vigour index decreased the most—by 17.8% for the female parent (*P* < 0.01) (1A) and by 26.8% for the male parent (*P* < 0.01) (2A).

### Effects of ionizing radiation on α-amylase activity

**Figure 2:**
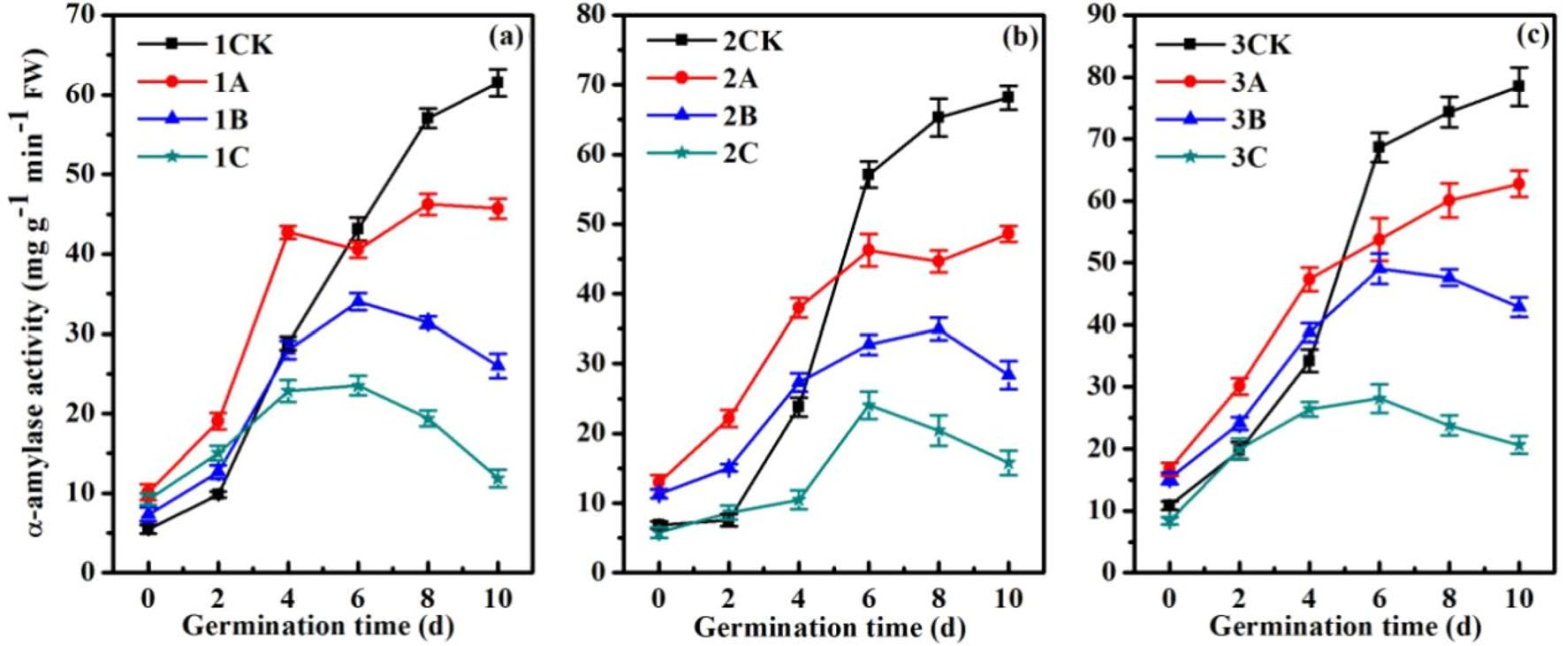
α-Amylase activity of rice seeds as a function of time in (**a**) XD10-01 (♀), (**b**) BX10 (♂), and (**c**) XJ (04)-08 under various doses of N ion beam irradiation. The means (± SEs) were calculated from three replicates per treatment.

During rice seed germination, the response of α-amylase activity to different doses of ionizing radiation differed (Fig. 2). According to the curves, we can conclude that the α-amylase activity of the high-dose irradiation group was markedly inhibited (1C, 2C, 3C). In the control group (1CK, 2CK, 3CK) and low-dose irradiation group (1A, 2A, 3A), the α-amylase activity increased gradually with increased cultivation time. Notably, the α-amylase activity of the three rice material control groups peaked on the 10th day (1CK, 2CK, 3CK).

The α-amylase activity markedly increased within 0–4 days in the low-dose irradiation groups compared with the control group but sharply decreased after 6–10 days (1A, 2A, 3A). Moreover, the α-amylase activity of the tetraploid rice decreased by 20% on the 10th day of cultivation (3A), while that of the female parent (1A) and male parent (2A) decreased by 25.7% and 28.7%, respectively.

The α-amylase activity of the medium-dose irradiation group (1B, 2B, 3B) increased within 0–4 days compared with that of the control group (1CK, 2CK, 3CK), but the increase was not as significant as that of the low-dose radiation group (1A, 2A, 3A). The α-amylase activity of the medium-dose irradiation group decreased markedly within 6–10 days of cultivation (1B, 2B, 3B). Moreover, the α-amylase activity of the medium-dose radiation group (1B, 2B, 3B) and the high-dose irradiation group (1C, 2C, 3C) tended to first increase and then decrease within 0–10 days of cultivation.

### Effects of ionizing radiation on soluble protein and carbohydrate contents

**Figure 3:**
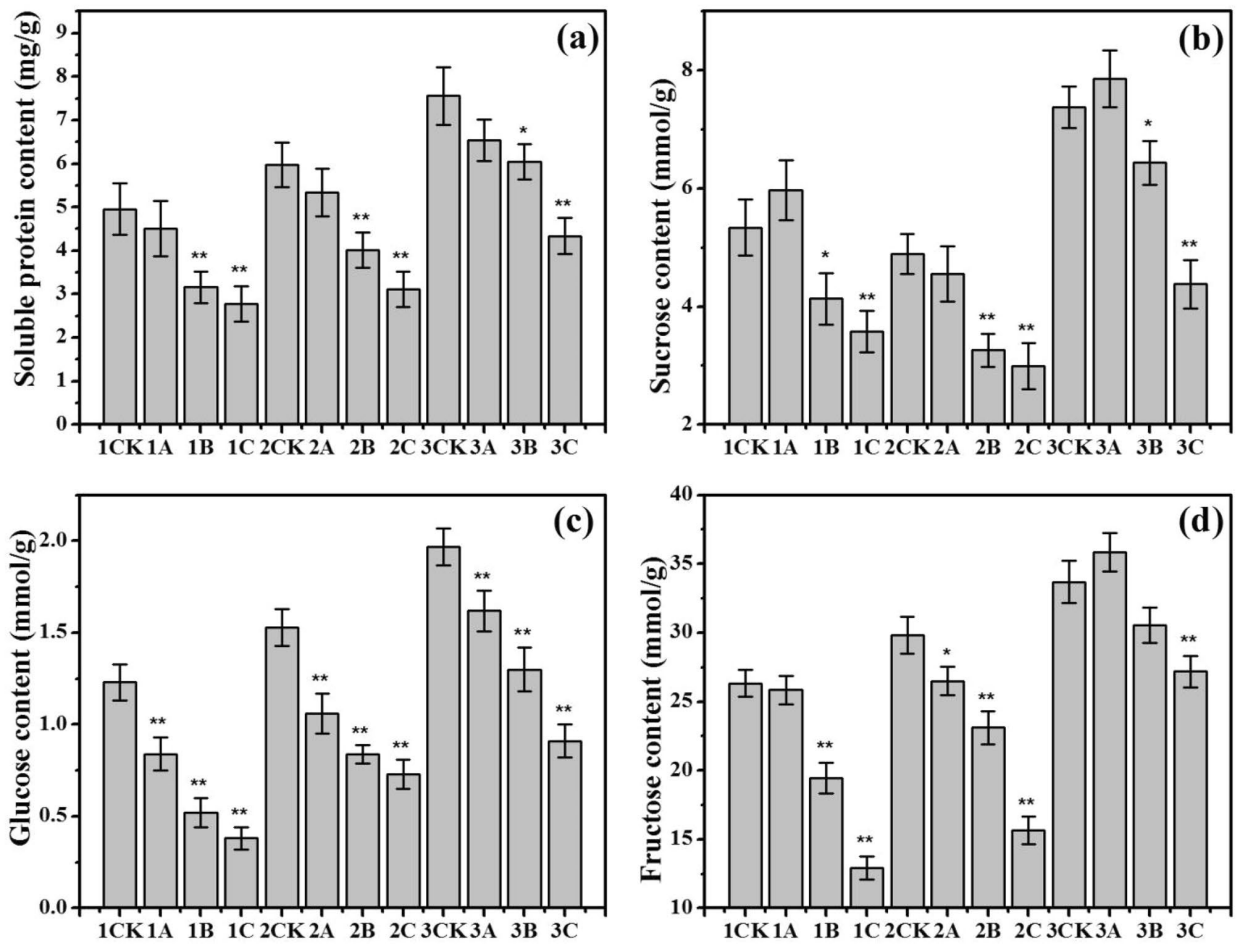
(**a**) Soluble protein content, (**b**) sucrose content, (**c**) glucose content, and (**d**) fructose content in rice seeds after being subjected to different doses of N ion beam irradiation. The means (± SEs) were calculated from three replicates per treatment. * and ** denote significances at the *P* < 0.05 and *P* < 0.01 levels, respectively, compared with the control group.

The soluble protein content and carbohydrate content of the tetraploid rice and its parents varied with increasing ionizing radiation (Fig. 3). Figure 3 shows that the soluble protein content and carbohydrate content of the tetraploid rice control group (3CK) were higher than those of the parent control groups (1CK, 2CK). Furthermore, compared with those of the female parent (1CK) and male parent (2CK) rice, the soluble protein content of the tetraploid rice increased by 52.7% and 26.6%, respectively, and the carbohydrate content increased by 13.1–60.2% (3CK).

As shown in Fig. 3a and c, the soluble protein content and glucose content of the tetraploid rice and its parents decreased with increasing irradiation dose, and the glucose content decreased markedly (*P* < 0.01). It is thus clear that the sucrose and fructose contents increased in response to low-dose ionizing radiation, as shown in Fig. 3b and d. Furthermore, the sucrose content and fructose content of the tetraploid rice subjected to low-dose ionizing radiation increased by 6.5% and 6.3%, respectively (3A), compared to those of the control rice, and the sucrose content of the female parent increased by 11.8% (1A).

### Effects of ionizing radiation on GA_3_ content

**Figure 4:**
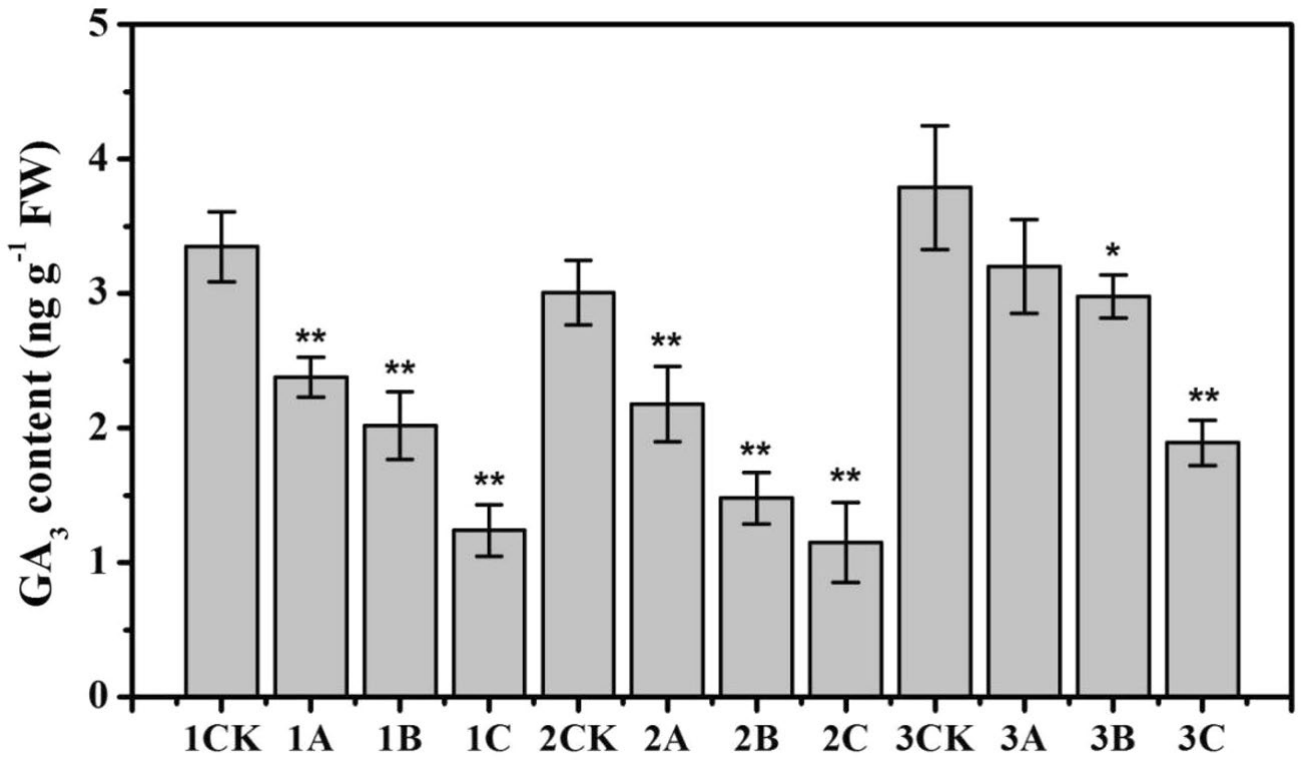
GA_3_ content in rice subjected to different doses of N ion beam irradiation. The means (± SEs) were calculated from three replicates per treatment. * and ** denote significance at the *P* < 0.05 and *P* < 0.01 levels, respectively, compared with the control group.

Among the various gibberellins, GA_3_ has vigorous physiological activity. GA_3_ can markedly promote the growth of plant stems and leaves, especially with respect to dwarf plants resulting from genetic and physiological factors. The GA_3_ content in the 5-day-old rice shoots subjected to different doses of ionizing radiation was determined to evaluate GA_3_-related expression and response to ionizing irradiation. As shown in Fig. 4, ionizing radiation could affect the GA_3_ content, and the GA_3_ content of both the tetraploid rice and the parents tended to decrease with increasing radiation dose. Moreover, the GA_3_ content in the female parent (1A) and male parent (2A) of the low-dose irradiation groups decreased by 29% and 27.6%, respectively (*P* < 0.01), compared to that of the control, while that in the tetraploid rice decreased by only 15.6% (*P* > 0.05) (3A). However, the GA_3_ content in both the tetraploid rice (3C) and its parents (1C, 2C) in the high-dose irradiation groups decreased markedly (*P* < 0.01). Taken together, these results suggest that the tolerance of the tetraploid rice to low-dose ionizing radiation is better than that of its parents.

### Response of the expression of essential genes related to α-amylase

**Figure 5:**
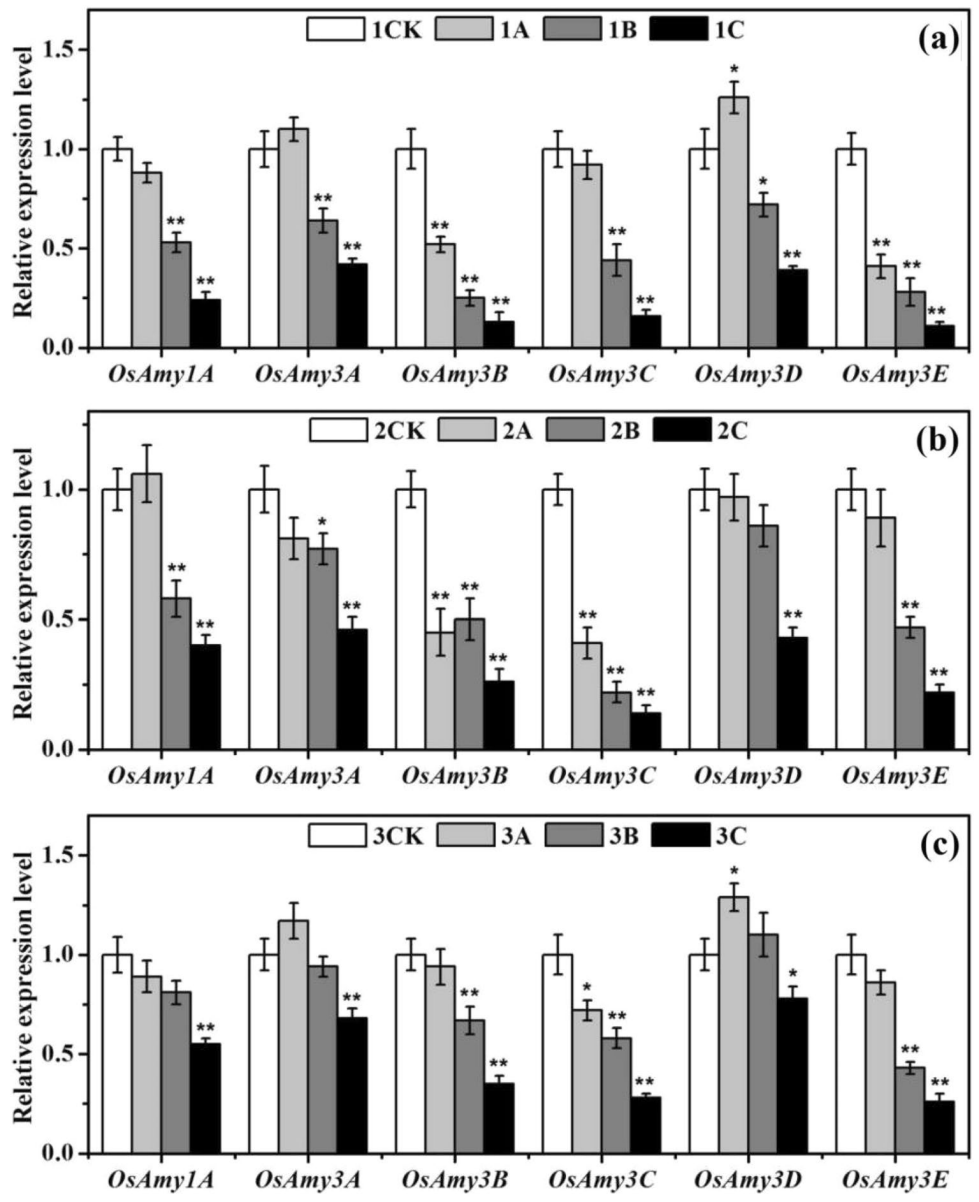
qPCR analysis of the key α-amylase genes (*OsAmy1A*, *OsAmy3A*, *OsAmy3B*, *OsAmy3C*, *OsAmy3D*, *OsAmy3E*) in the nonirradiated and irradiated groups whose plants were subjected to different doses of ionizing radiation. The results are reported as the relative expression of each gene transcript with respect to an internal standard. The relative value of the expression of each gene in the nonirradiated sample was set to 1. The vertical bars represent the means ± SEs of three independent biological replicates (*n* = 3). * and ** denote significance at the *P* < 0.05 and *P* < 0.01 levels, respectively, compared with the control group; (**a**), (**b**), and (**c**) represent XD10-01 (♀), BX10 (♂), and XJ (04)-08, respectively.

Ionizing radiation can disrupt the regulation and expression of key α-amylase genes during rice seed germination. To study the biological effects of ionizing radiation on tetraploid rice in detail, the expression levels of *OsAmys* α-amylase genes (*OsAmy1A*, *OsAmy3A*, *OsAmy3B*, *OsAmy3C*, *OsAmy3D*, *OsAmy3E*) in 5-day-old rice shoots subjected to different does of ionizing radiation were measured. Figure 5 shows that the regular expression of these six α-amylase genes in rice was affected by the ionizing radiation.

For the tetraploid rice subjected to low-dose ionizing radiation, both *OsAmy3A* and *OsAmy3D* were upregulated compared to those in the control, between which *OsAmy3D* was upregulated by 1.29 times (*P* < 0.05), and the other four genes were downregulated, among which *OsAmy3C* was downregulated by 0.72 times (*P* < 0.05) (3A). For the female parent, both *OsAmy3A* and *OsAmy3D* were upregulated, between which *OsAmy3D* was upregulated by 1.26 times (*P* < 0.05), and four genes were downregulated, among which *OsAmy3B* and *OsAmy3E* were downregulated by 0.52 and 0.41 times, respectively (*P* < 0.01) (1A). For the male parent, five genes were downregulated, among which *OsAmy3B* and *OsAmy3C* were downregulated by 0.45 and 0.41 times, respectively (*P* < 0.01), and *OsAmy1A* was not markedly upregulated (*P* > 0.05) (2A).

There were three genes downregulated in the medium-dose-irradiated tetraploid rice subspecies compared to the control rice (*P* < 0.01) (3B), while there were five genes downregulated in the female parent (1B) and four downregulated in the male parent (2B). Accordingly, the degree of inhibition in the parents was markedly higher than that in the tetraploid rice.

### Response of the expression of essential genes related to gibberellin metabolism

**Figure 6:**
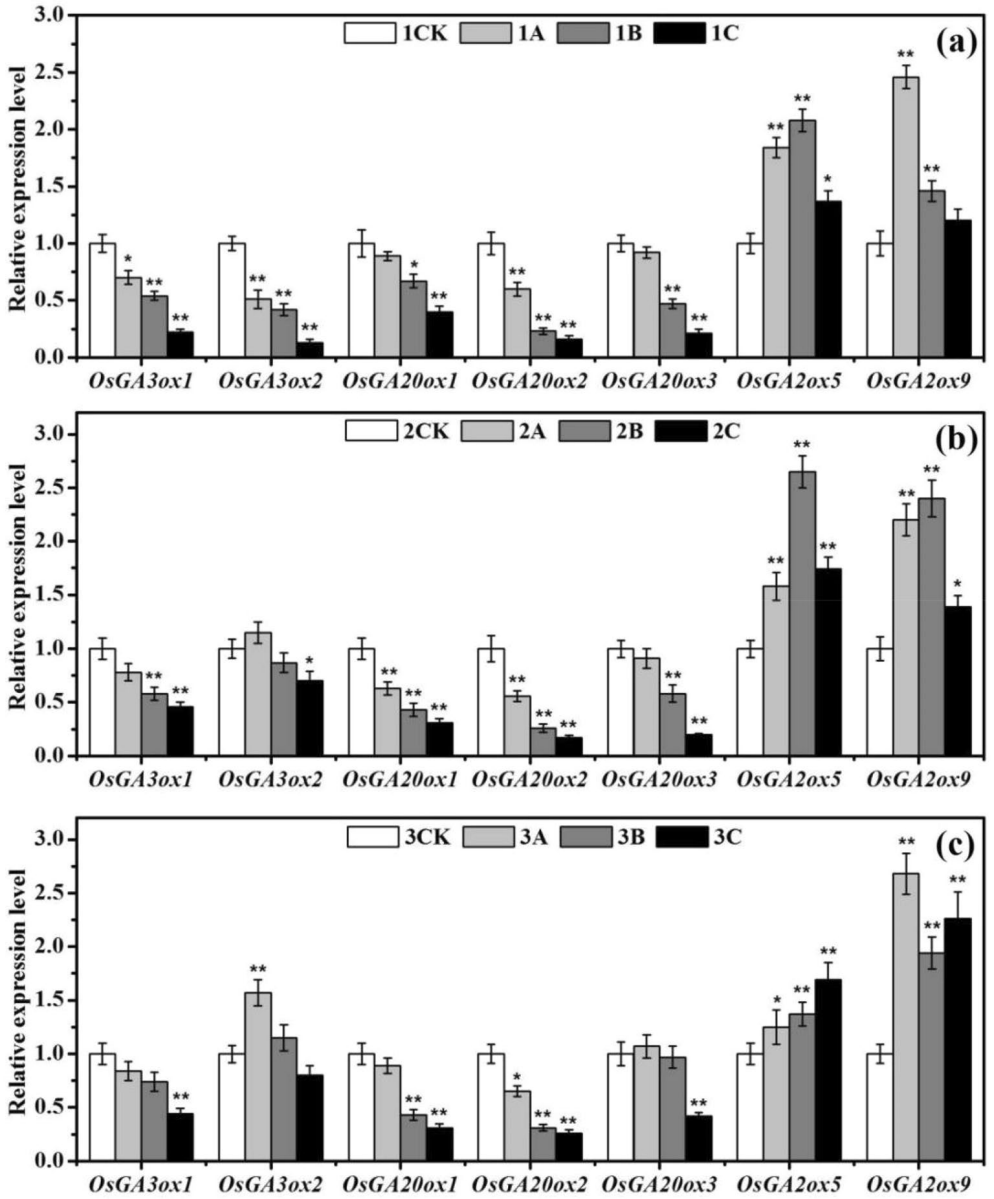
qPCR analysis of the key genes involved in gibberellin metabolism (*OsGA3ox1, OsGA3ox2, OsGA20ox1, OsGA20ox2, OsGA20ox3, OsGA2ox5, OsGA2ox9*) in the nonirradiated and irradiated groups subjected to different doses of ionizing radiation. The results are reported as the relative expression of each gene with respect to an internal standard. The relative value of the expression of each gene in the nonirradiated sample was set to 1. The vertical bars represent the means ± SEs of three independent biological replicates (*n* = 3). * and ** denote significance at the *P* < 0.05 and *P* < 0.01 levels, respectively, compared with the control group; (**a**), (**b**), and (**c**) represent XD10-01 (♀), BX10 (♂), and XJ (04)-08, respectively.

Ionizing radiation can disrupt the regulation and expression of gibberellin metabolism-related genes during rice seed germination. To study the biological effects of ionizing radiation on tetraploid rice, the expression of essential genes involved in gibberellin biosynthesis (*OsGA3ox1*, *OsGA3ox2*, *OsGA20ox1*, *OsGA20ox2*, *OsGA20ox3*) and catabolism (*OsGA2ox5*, *OsGA2ox9*) was measured in 5-day-old rice shoots subjected to various doses of ionizing radiation. Figure 6 shows that the regular expression of the seven gibberellin metabolism-related genes in rice was affected by ionizing radiation; notably, the catabolism-related genes were markedly upregulated. With increasing ionizing radiation dose, the gibberellin catabolism-related gene *OsGA2ox5* of the tetraploid rice subspecies tended to gradually increase, and *OsGA2ox9* tended to first increase, decrease, and then increase. In contrast, the expression of the two gibberellin catabolism-related genes of the parents tended to first increase and then decrease.

The responses of the tetraploid rice subjected to different doses of ionizing radiation were different from those of its parents. Most notably, the expression of critical genes involved in gibberellin synthesis under low-dose irradiation was inhibited. Moreover, the degree of inhibition of the tetraploid rice (3A) was lower than those of its parents (1A, 2A). Compared with that of the control rice, the expression of the gibberellin biosynthesis-related gene *OsGA3ox2* of the tetraploid rice was upregulated by 1.57 times (*P* < 0.01), and that of *OsGA20ox3* was upregulated by 1.07 times (3A); additionally, the expression of *OsGA3ox2* and *OsGA20ox2* was downregulated (*P* < 0.01) and that of *OsGA3ox1* was downregulated (*P* < 0.05) in the female parent (1A), and the expression of *OsGA20ox1* and *OsGA20ox2* was downregulated (*P* < 0.01) in the male parent (2A). Thus, we can see that the degree of inhibition in the parents is markedly higher than those in the tetraploid rice.

## Discussion

Taking advantage of polyploidization, which leads to increased sizes of plant organs and heterozygosity, researchers have developed new plant varieties with increased yields, improved quality and strong stress resistance^30–32^. Tetraploid *Carrizo citrange* rootstock showed a smaller decrease in photosynthetic rate and starch levels and lower levels of MDA and electrolyte leakage than did diploids during the coldest months^33^. Under salt stress, compared with its diploid counterpart, tetraploid *Robinia pseudoacacia* L. maintained a completely intact structure. The accumulation of hydrogen peroxide (H_2_O_2_), necessary antioxidant enzymes and nonenzymatic antioxidants in tetraploid plants was shown to be greater than that in diploid plants^34^. Tetraploid black locust is more tolerant to salt stress than is diploid black locust, and methyl jasmonate has been shown to play a role in the defence response^35^. Tetraploid chromosomes are hypomethylated, and the genomic loci related to stress-responsive genes are enhanced; thus, tetraploid rice is more salt tolerant than diploid rice is^36^. Moreover, the novel haplotype *HSP101-1* plays a substantial role in both seedling thermal stress resistance and the total fertility rate of neotetraploid rice^37^.

In this work, we studied the growth response, specifically during seed germination, of *indica–japonica* tetraploid rice to low-dose ionizing radiation. Chromosome doubling resulted in a sharp increase in the expression of genes, so the α-amylase, soluble proteins and sugars produced during germination also increased markedly, which could help in the acquisition of more nutrients for seed germination and seedling growth (Figs. 2, 3). There are two kinds of genomes in *indica–japonica* tetraploid rice, namely, an *indica* one and a *japonica* one, which present strong heterosis. Table 1 shows that the germination rate and seedling height of *indica–japonica* tetraploid rice under low-dose irradiation decreased statistically insignificantly (*P* > 0.05) (3A), while those of the parents decreased markedly (1A, 2A). The reason for this result may because the expression of *OsGA3ox2* was upregulated by 1.57 times (*P* < 0.01) and because the expression of *OsGA20ox3* was upregulated by 1.07 times, resulting in the statistically insignificant decrease in the GA_3_ content of the *indica–japonica* subspecies rice (*P* > 0.05) (3A), while those in both parents decreased markedly (*P* < 0.01) (1A, 2A) (Fig. 4). Moreover, the expression of *OsAmy3D* was upregulated by 1.29 times (*P* < 0.05); the expression of *OsAmy3A* was upregulated by 1.17 times; and the expression of *OsAmy1A*, *OsAmy3B* and *OsAmy3E* was not markedly regulated (*P* > 0.05). This resulted in the α-amylase activity gradually increasing with increasing cultivation time (3A), while this activity in both parents decreased (1A, 2A) (Fig. 2). Due to the abundance of α-amylase and gibberellin, the seed water absorption rate of the tetraploid rice showed no significant difference compared with that of the control rice from 0 to 36 h (Fig. 1) (3A). Only when the seeds absorb enough water can the nutrients stored in the cotyledons or endosperm be transformed into soluble proteins and sugars, which provide the basis for embryo development. In this study, the sucrose content and fructose content in the tetraploid rice subjected to low-dose ionizing radiation increased by 6.5% and 6.3% (3A), respectively, compared with those in the control rice (3CK) (Fig. 3). Only when an embryo has sufficient nutrients can it also have strong seed vigour. The seedlings grew tall, and the root system developed (3A) (Table 1). This conclusion is consistent with those of existing studies. Wang et al. argued that the decrease in active gibberellin content in rice seeds under low temperature leads to a decline in seed sugar consumption and seed germination rate^38^. Furthermore, salt stress leads to a lack of bioactive gibberellin, so α-amylase gene expression was downregulated, and α-amylase activity was inhibited. Finally, the germination rate of rice seeds was reduced^39^.

The biological effect of low-dose ionizing radiation on rice, other crop species and microbes has been determined via genetic studies beginning in the 1980s. Mutation breeding of rice via ionizing radiation results in high mutation rates and broad-spectrum mutations, and many different mutations can be induced^40,41^. The effects of ionizing radiation on rice morphology, anatomy, biochemistry, and biology was dose dependent^11,42^. In this study, N^+^ ion irradiation at three different doses reduced the relative water absorption by, seed vigour of and GA_3_ content in rice seeds (Figs. 1, 4; Table 1). The degree of inhibition gradually increased with increasing irradiation dose. The dose of ionizing radiation also had a significant effect on the germination rate of *Medicago truncatula* seeds, which is consistent with our results^43^.

There is growing evidence that ionizing radiation can help promote resistance to adverse environmental damage by activating the plant’s own antioxidant defence system, reducing the H_2_O_2_ content, hydroxyl radical production, the generation of superoxide anions and the MDA content^44^. In our previous study, low-dose ionizing radiation increased the antioxidant enzyme activity in seedlings of *indica–japonica* tetraploid rice and its parents and increased the content of carotenoids, which act as antioxidants^45,46^. In the present study, low-dose ionizing radiation markedly increased the α-amylase activity in *indica–japonica* tetraploid rice and its parents within 0–4 days of cultivation, which improved the adaptability of the plants to stress conditions (1A, 2A, 3A). In contrast, high-dose ionizing radiation severely decreased the water absorption by the seeds and the seed vigour, inhibited α-amylase activity, upregulated the expression of critical genes involved in gibberellin catabolism (Fig. 6), and ultimately inhibited seed germination (Table 1) (1C, 2C, 3C). High-dose ionizing radiation is therefore unfavourable to the growth and development of rice plants. This biological effect of ionizing radiation is defined as a radiation stimulatory effect. That is, low-dose ionizing radiation has a stimulatory effect, while high-dose ionizing radiation has an inhibitory effect^47^.

**Figure 7:**
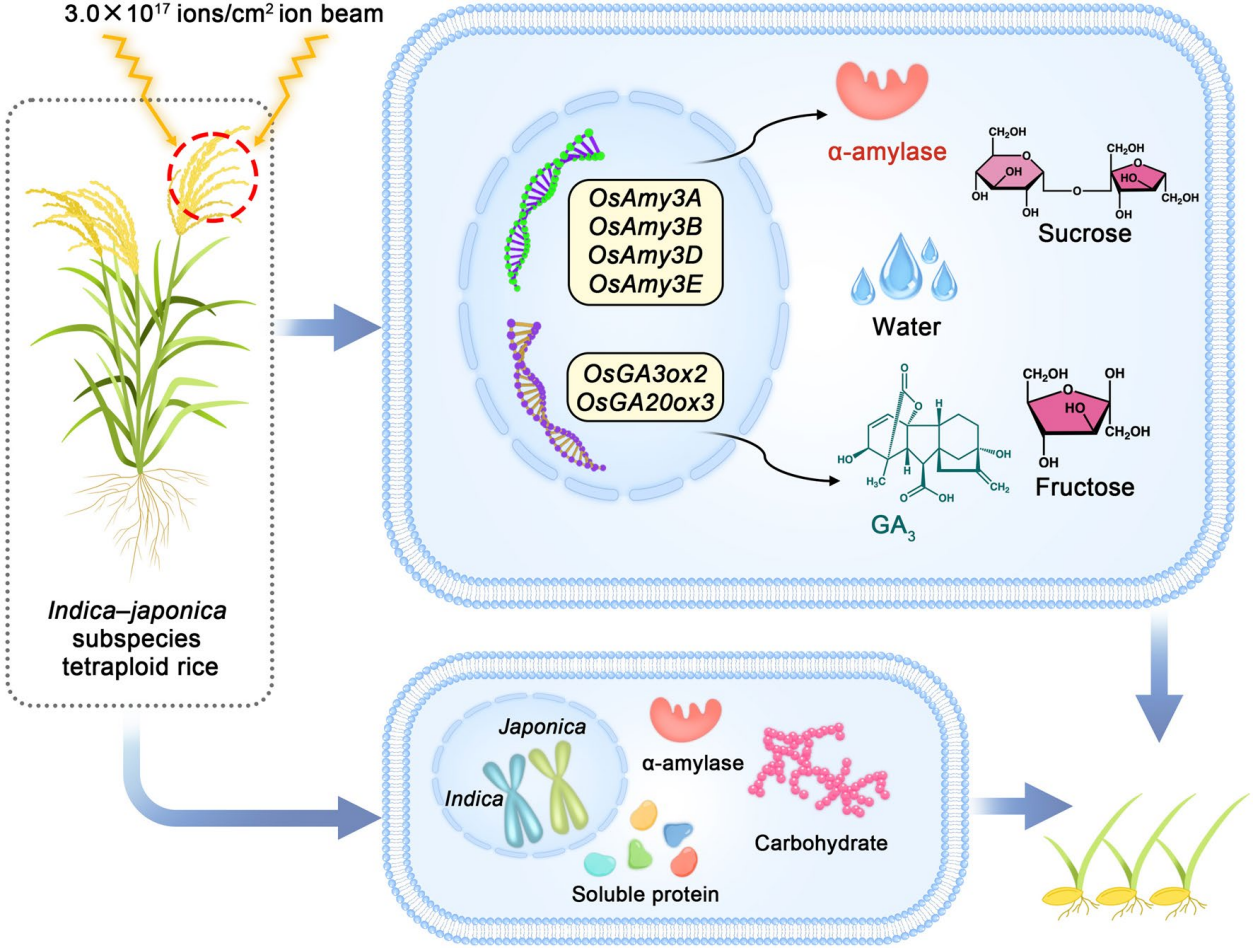
Diagram of the network at play in response to 3.0 × 10^17^ ions/cm^2^ N^+^ ion beam irradiation in *indica–japonica* tetraploid rice. The figure shows that under low-dose ionizing radiation, the expression levels of some α-amylase genes and gibberellin synthesis-related genes in tetraploid rice were higher than those in the parents, resulting in the tetraploid rice having higher α-amylase activity and GA_3_ content. Thus, the tetraploid rice has a higher water absorption rate than its parents have, which would lead increased production of soluble proteins and carbohydrates (via degradation), and the contents of sucrose and fructose are higher than those in the control rice. At the same time, the doubling of the genome enabled tetraploid rice to produce higher levels of α-amylase, soluble proteins and carbohydrates than the diploid parents produced. These two reasons jointly determine that the germination rate and seedling height of the tetraploid rice decreased nonsignificantly compared with those of the control, while those of the diploid parents decreased significantly under low-dose ionizing radiation.

Plants are inevitably impacted by various stresses since they are exposed to environmental conditions and cannot move. Nonetheless, throughout their systematic evolution, plants have established effective adaptation and even resistance mechanisms. In this study, we investigated the regulation of the expression of genes involved in α-amylase synthesis and gibberellin metabolism in plants subjected to ionizing radiation. The results showed that the number and degree of inhibited genes of the tetraploid rice were lower than those of its parents under three different doses of ionizing radiation. This work provides reference data for elucidating the molecular mechanism through which ionizing radiation affects plant biology. However, the limitations of this study are apparent. The analysis of expression levels of genes related to α-amylase synthesis and gibberellin metabolism during seed germination can only partly clarify the biological effect of ionizing radiation. To further understand the formation of this stress resistance, transcriptome analysis is also needed, which is highly important to agricultural production. Finally, on the basis of our data, we constructed a representative model of the network at play in response to 3.0 × 10^17^ ions/cm^2^ N^+^ ion beam irradiation in *indica–japonica* tetraploid rice (Fig. 7).

## Conclusion

In brief, recent research has revealed the stress responses of tetraploid rice by elucidating the physiological and molecular responses through which seeds of *indica–japonica* tetraploid rice germinate under ionizing radiation. We found that 7.0 × 10^17^ ions/cm^2^ irradiation dramatically decreased seed water absorption, seed vigour and α-amylase activity and interfered with the expression of genes related to α-amylase synthesis and gibberellin metabolism. Under 3.0 × 10^17^ ions/cm^2^ irradiation, with respect to *indica–japonica* tetraploid rice, the upregulation of *OsGA3ox2* and *OsGA20ox3* resulted in an increase in GA_3_ content. Similarly, the upregulation of *OsAmy3A* and *OsAmy3D* resulted in sharp increase in α-amylase activity, the water absorption rate, and sucrose and fructose contents, which resulted in the seed vigour being higher than that of its parents. Our findings lay a foundation for further research on the physiological and molecular features of *indica–japonica* tetraploid rice seed germination in response to stress.

## Materials and methods

### Test materials

The test materials include the parent materials XD10-01 (*japonica* female parent, number 1) and BX10 (*indica* male parent, number 2) and the *indica–japonica* tetraploid rice subspecies XJ (04)-08 (number 3). XJ (04)-08 is a stable tetraploid line produced from calli of a diploid *indica–japonica* F_1_ hybrid obtained from a XD10-01 × BX10 cross after chromosome doubling by colchicine and tissue culture.

### Ionizing radiation

Mature dry seeds whose embryo faced upwards were subjected to 30 keV N^+^ beams via a multifunctional ion injector (Chengdu, Tongchuan, China). The radiation doses were 0 × 10^17^ (CK), 3.0 × 10^17^ (A, low dose), 5.0 × 10^17^ (B, medium dose), and 7.0 × 10^17^ ions/cm^2^ (C, high dose). Twelve different groups of plants were treated for subsequent experiments. Each treatment group included 3 replicates, with 100 seeds per replicate.

### Relative water absorption of seeds

In each group, 100 seeds were randomly selected, and their dry weight was determined. Then, they were put into a beaker, and 100 mL of distilled water was added for the seeds to imbibe. After 3, 6, 9, 12, 18, 24, 30, 36, and 48 h, the seeds were removed and weighed again. The relative water absorption was calculated as (B − A)/A × 100%, where A and B are the seed quality before and after the water absorption, respectively.

### Seed vigour and seedling growth indexes

The rice seeds were soaked in distilled water for 24 h, sterilized with 0.1% mercuric chloride for 15 min, washed with distilled water 3 times and then placed on a single layer of filter paper in a Petri dish. After 3 days of incubation in the dark at 28 °C, the seedlings were transferred to a light incubator at 28 °C for cultivation, and the germination percentage was determined. The germination percentage of each group was evaluated every 24 h for 6 days. The seedling height, root (> 0.5 cm) number and root length (the root with the most root hairs) were measured on the 7th day. The calculation formulas used were as follows:

$$ {\text{Germination}}\;{\text{rate }}\;\left( \% \right) \, = {\text{ Number}}\;{\text{ of}}\;{\text{ seeds}}\;{\text{ germinated }}/{\text{ Total}}\;{\text{ number }}\;{\text{of}}\;{\text{seeds}} \times {1}00 $$

$$ {\text{Germination }}\;{\text{index }} = \sum {\frac{Gt}{{Dt}}} ({\text{Gt}}:{\text{Number}}\;{\text{ of}}\;{\text{ germinated }}\;{\text{seeds }}\;{\text{at}}\;{\text{ different}}\;{\text{ time }}\;{\text{points}}; \, {\text{Dt}}:{\text{Germination }}\;{\text{days}}); $$

$$ {\text{Vigour }}\;{\text{index}} = {\text{ Germination }}\;{\text{index}} \times {\text{Average}}\;{\text{ dry }}\;{\text{weight }}\;{\text{of}}\;{\text{a}}\;{\text{single }}\;{\text{seedling}} $$

### α-Amylase activity

The a-amylase activity was determined using 3,5-dinitrosalicylic acid. Seedlings (1 g) cultivated under light for 0, 2, 4, 6, 8 and 10 days were put into a mortar, to which a small amount of quartz sand and 10 mL of 0.2 M phosphate buffer solution (pH 5.8) were added. The mixture was ground into a homogenate and subsequently centrifuged at 5000 r/min for 10 min. The supernatant was brought to 100 mL. There were 2 test tubes per replicate—a control tube and a determination tube. One millilitre of enzyme solution was added to each tube, which were then transferred to a 70 °C water bath for 15 min to inhibit the activity of β-amylase. Then, the tubes were removed and cooled under running water. Two millilitres of 3,5-dinitrosalicylic acid was added to each replicated control tube, which were then placed in a water bath at 40 °C for 10 min. One millilitre of 1% starch solution preheated to 40 °C was subsequently added to each test tube, after which the tubes were then placed in a 40 °C water bath for 5 min after being shaking, and then, 2 mL of 3,5-dinitrosalicylic acid was added to each determination tube, which were subsequently shaken. Each tube was placed in a boiling water bath for 10 min and then allowed to cool. Distilled water was added such that the volume of each tube was brought to 20 mL. The activity of α-amylase was measured after the tubes were shaken.

### Soluble protein content

A total of 0.5 g of 5-day-old rice buds was cultivated in the light and put into a mortar. Citric acid buffer (pH 5.6) was added, after which the samples were ground into homogenate. The homogenate was subsequently poured into a 10 mL centrifuge tube, fixed in buffer solution, and centrifuged at 4000 r/min for 15 min at 4 °C. The supernatant was taken for analysis. A total of 1.0 mL of the sample extract was put into a test tube, after which 5 mL of Coomassie brilliant blue reagent was added. The tube was shaken and then incubated at room temperature for 2 min. The absorbance was determined at a wavelength of 595 nm, and the soluble protein content was calculated according to a standard curve.

### Carbohydrate content

Five-day-old rice buds were dried for 15 min at 110 °C and then incubated at 70 °C overnight. The dry matter was subsequently ground in a mortar. Fifty milligrams of the sample was weighed and transferred to a 10 mL centrifuge tube. Then, 4 mL of 80% ethanol was added. The samples were subsequently placed in an 80 °C water bath for 40 min, and the supernatant was collected after centrifugation. Afterwards, 2 mL of 80% ethanol was added to the residue; then, the samples were extracted twice, and the supernatants were combined. The combined supernatant was added to 10 mg of activated carbon and decolorized at 80 °C for 30 min. The volume was subsequently brought to 10 mL with 80% ethanol, and the extracted solution was obtained after filtration.

To determine the sucrose content, 0.4 mL of extraction solution was taken, after which 200 μL of 2 mol/L NaOH was added. The solution was boiled at 100 °C for 5 min and then allowed to cool. Afterwards, 2.8 mL of 30% HCl and 0.8 mL of 0.1% resorcinol were added, the solutions were shaken thoroughly, and then they were allowed to react in a water bath at 80 °C for 10 min. Finally, the OD value at 480 nm was measured after the solution cooled, and the sucrose content was calculated according to a standard curve.

To determine the glucose content, four millilitres of enzyme preparation was added to a test tube, which was then placed in a 30 °C water bath. After the temperature of the enzyme solution in the tube reached equilibrium, 2 mL of extraction solution was added, and the tube was shaken thoroughly; the temperature was maintained for 5 min, after which 8 mL of 10 mol/L sulfuric acid was added to stop the reaction. When the solution reached room temperature, the OD value at 460 nm was measured, and the glucose content was calculated according to the standard curve.

To determine the fructose content, 1 mL of extract was added to 2 mL of 30% HCl, 1 mL of 0.1% resorcinol and 1 mL of H_2_O. Afterwards, the tube was shaken thoroughly. The mixture was allowed to react in a water bath at 80 °C for 10 min, after which it was allowed to cool to room temperature. The OD value at 480 nm was subsequently measured.

### GA_3_ content

Rice seedlings (0.5 g) cultivated in the light for 5 days were ground to a powder in liquid nitrogen in an ice bath, and then 3 mL of 80% methanol was added while the homogenate was being ground, which was then transferred to a test tube. The content of GA_3_ was measured via a gibberellin enzyme-linked immunosorbent assay (ELISA) kit produced by Shanghai Enzyme Linked Biotechnology Co., Ltd.

### RNA isolation and quantitative polymerase chain reaction (qPCR)

Five-day-old rice buds from each treatment group were collected, frozen in liquid nitrogen and then stored at − 80 °C for RNA extraction. The extraction was performed via TRIzol reagent according to the instructions of Invitrogen, the manufacturer. The total RNA extracted was tested for integrity and purity via 1% agarose gel electrophoresis without RNase contamination. cDNA was synthesized with a PrimeScript™ RT‒PCR Kit according to the manufacturer’s instructions. The primers used for quantitative analysis were designed by staff at Shanghai Meiji Biomedical Technology Co., Ltd. A Quantitative Studio Three Real-Time qPCR system and DyNAmo™ SYBR Green qPCR kit were used for fluorescent real-time qPCR. The quantitative PCR conditions were as follows: 95 °C for 10 min, followed by 40 cycles of 95 °C for 30 s; 60 °C for 30 s; and 72 °C for 20 s. Afterwards, a dissolution curve was constructed. CFX Manager software was used to collect and process the data, and the Ct(2^−ΔΔCt^) approach was used to compute the relative expression of objective genes and reference genes. The *OsActin* gene was used as an internal reference, and the experiments were repeated three times.

### Statistical analysis

All the data were evaluated using IBM SPSS statistical software (version 19). One-way ANOVA was used to determine the significance of the outcomes between the irradiated group and nonirradiated group. The standard error (SE) is indicated as an error bar in the graphs and as a ± symbol in the tables.

## Data Availability

All data generated or analyzed during this study are included in this published article.

## Abbreviations

GA_3_: Gibberellic acid 3
FAO: Food and Agriculture Organization of the United Nations
IAEA: International Atomic Energy Agency
LET: Linear energy transfer
DNA: Deoxyribonucleic acid
cDNA: Complementary DNA
RNA: Ribonucleic acid
ABA: Abscisic acid
MDA: Malondialdehyde
CAT: Catalase
PCR: Polymerase chain reaction
keV: Kiloelectron volt
RT-PCR: Reverse transcription-polymerase chain reaction
OD: Optical density
SE: Standard error
ANOVA: Analysis of variance
